# What is the most fixable intramedullary implant for basicervical fracture and transcervical shear fracture? – A finite element study

**DOI:** 10.1016/j.jcot.2022.102015

**Published:** 2022-09-20

**Authors:** Motoharu Komatsu, Takehiro Iwami, Hiroaki Kijima, Tetsuya Kawano, Naohisa Miyakoshi

**Affiliations:** a Graduate School of Engineering Science, Akita University, Japan; b Department of Orthopedic Surgery, Akita University Graduate School of Medicine, Akita, Japan; c Akita Hip Research Group (AHRG), Akita, Japan

**Keywords:** Finite element analysis, Proximal femoral fractures, Intramedullary fixation, Basicervical fracture, Transcervical shear fracture

## Abstract

**Objective:**

The objectives of this study are 1) to biomechanically compare six different intramedullary fixations for basicervical fracture (AO 31-B3, Type 2 in area classification) and transcervical shear fracture (AO 31-B2.3, Type 1–2 in area classification) using the finite element (FE) method, and 2) to investigate the effects of two different unstable fracture types on fixation.

**Methods:**

FE models of two different types of proximal femoral fractures are constructed from CT scan images of a patient with osteoporosis. The fracture models are fixed with a short femoral nail with a single lag screw, short femoral nail with a single blade, and short femoral nail with double lag screws, and then fixed with long femoral nails for each of the three nail types. Subsequently, the maximum loads during walking and stair climbing, as well as the minimum principal strain and compressive failure elements are calculated to assess the fixation of each implant.

**Results:**

In both fracture types, the long nail with double lag screws show the smallest volume of compressive failure elements (basicervical fracture, 2 mm^3^; transcervical shear fracture, 217 mm^3^). In all types of implants, the volume of the compressive failure elements is larger in the transcervical shear fracture than in the basicervical fracture. A similar trend is observed for the minimum principal strain (compressive strain).

**Conclusion:**

The present study shows that a long nail with double lag screws is the most fixative intramedullary nail device for basicervical fracture and transcervical shear fracture in any condition. Furthermore, it is shown that transcervical shear fracture is considerably more unstable than basicervical fracture.

## 1 Introduction

In recent years, the proportion of elderly people in the world's total population as well as the incidence of proximal femoral fractures have increased.^1^^,^^2^ Various classification methods for these fractures have been devised to support treatment options for proximal femoral fractures.^3^, ^4^, ^5^, ^6^ However, because of the wide variety of classification methods, treatment options are diverse and remain non-unified.^7^ Therefore, to systematise treatment options, Kijima et al. introduced ‘area classification’, which is a comprehensive classification method for proximal femoral fractures.^8^

In area classification, or in the AO/OTA classification, which is widely used globally, the basicervical fracture (Type 2 in area classification, 31-B3 in AO classification) and transcervical shear fracture (Type 1–2 in area classification, 31-B2.3 in AO classification) exhibit particularly high instability.^9^^,^^10^ The basicervical fracture is defined as a fracture that occurs medial to the intertrochanteric line above the minor trochanter.^11^ Moreover, Deneca et al. reported that basicervical fracture have greater rotational instability than that associated with other fractures.^12^ Therefore, the intramedullary nails with double lag screws, including an anti-rotation screw, have been introduced to improve the rotational stability of basicervical fracture.^13^^,^^14^ Transcervical shear fractures, also called Pauwels type 3 fractures are prone to shearing force at the angle of the main fracture line. Intramedullary nails reportedly provide greater stability to such fractures than do 3 screws or extramedullary devices.^15^ For these reasons, we mainly treat the Pauwels type 3 fracture with intramedullary nails. Therefore, we investigated what type of intramedullary nails should be used for both types of fractures in this study. Intramedullary nail fixation is a treatment for these types of fractures. The advantages of intramedullary fixation include shorter operative time, less blood loss, and a higher failure load of the implant. Furthermore, it has been reported to be an effective treatment for unstable fractures.^16^, ^17^, ^18^ However, excessive shearing force on the fracture surface causes secondary displacement of bone fragments and, in extreme cases, complications such as ‘cut-out’. In particular, patients with osteoporosis whose central area of the femoral neck often lacks cancellous bone are likely to exhibit decreased fixation, thereby resulting in complications.^19^^,^^20^ Therefore, a strong fixation against shearing force must be ensured at the fracture points, and various types of intramedullary implants are available for this purpose. However, the fixation of intramedullary nails for basicervical and transcervical shear fractures is rarely investigated, and the selection of the optimal intramedullary implant for these fractures remains controversial.

To determine the most appropriate internal fixation device, fixation must be compared mechanically and quantitatively between devices for the same bone and fracture type. The objectives of this study are 1) to biomechanically compare six different intramedullary fixations for basicervical and transcervical shear fractures using the finite element (FE) method and 2) to investigate the effects of two different unstable fracture types on fixation.

## 2 Materials and methods

This study was approved by the Ethics Committee of the Akita University School of Medicine (Reception No. 2482).

### 2.1 FE models

The subject for the FE models was an osteoporosis patient (age: 73 years, height: 165 cm, weight: 66.2 kg). The patient was diagnosed with osteoporosis because of a proximal femoral fracture due to low-energy trauma. A CT scan (Revolution CT, GE Healthcare, USA) was performed on the subject's lower extremities based on slice thicknesses of 1.25 mm and 512 × 512 pixels per image. In addition, a bone mass phantom (QRM Quality Assurance in Radiology and Medicine GmbH, Baiersdorfer, Germany) was scanned. Table 1 shows the detailed data of bone mineral density obtained using quantitative CT (QCT), which confirmed that the subject was an osteoporotic patient. Mechanical Finder (version 11, Standard Edition) (Research Center of Computational Mechanics, Inc., Tokyo, Japan) was the software to evaluate bone strength, as well as to construct the FE model and perform FE analysis. A three-dimensional (3D) model of the intact femur was constructed by extracting the region of interest around the cortical bone from each CT scan image of the lower extremity that had not fractured.

**Table 1:** Bone mineral density measured using QCT and literature values^21^ (mg/cm^3^).

Volume of interest	Present study	Bousson et al., 2001 (QCT)	
		Hip fracture subjects (n = 47), mean (SD)	Contrals (n = 60), mean (SD)
Femoral head	199.4	182.2 (44.7)	237.1 (52.3)
Femoral neck	260.1	242.5 (48.7)	291.5 (48.3)

**Fig. 1:**
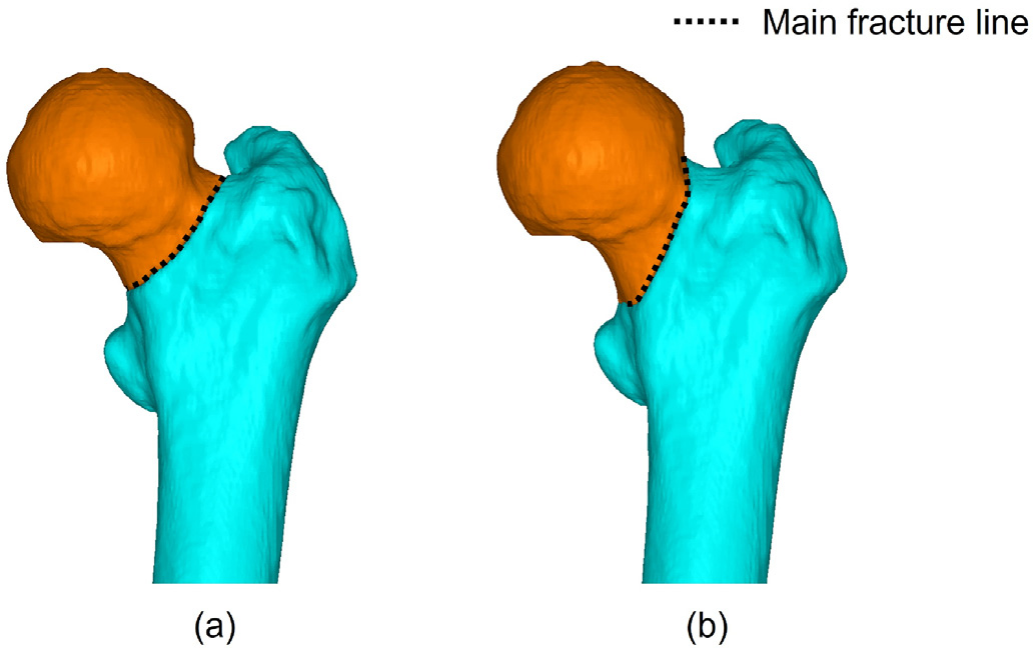
Positions of main fracture line: (a) Basicervical fracture (AO 31-B3, Type 2 in area classification); (b) transcervical shear fracture (AO 31-B2.3, Type1-2 in area classification).

**Fig. 2:**
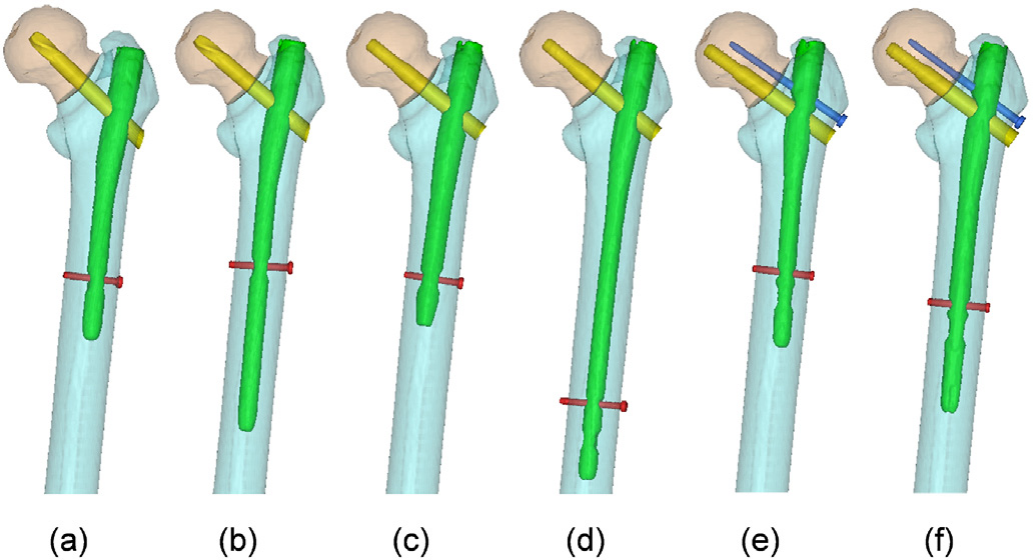
3D model with implants inserted in Type 2: (a) Short nail of Group 1; (b) long nail of Group 1; (c) short nail of Group 2; (d) long nail of Group 2; (e) short nail of Group 3; (f) long nail of Group 3. Group 1: one blade; Group 2: one lag screw; Group 3: double lag screws.

Next, based on area classification,^8^ the fracture line of the basicervical fracture (Type 2 in area classification, 31-B3 in AO classification) and transcervical shear fracture (Type 1–2 in area classification, 31-B2.3 in AO classification) were applied to the 3D femur model (Fig. 1). To fix these fractures, six types of intramedullary implants were used. We classified the intramedullary nail fixation modes into three groups based on the device to be inserted into the femoral head: Trochanteric Fixation Nail Advanced (TFNA) with a single blade (DePuy Synthes, Warsaw, IN, USA) (Group 1), Gamma 3 nail with a single lag screw (Stryker, Mahwah, NJ, USA) (Group 2), and Cephalomedullary Asia nail with double lag screws (Zimmer Biomet, Warsaw, IN, USA) (Group 3); short and long nails were used for each group (Fig. 2). The 3D models of each nail were constructed using the same method as the bone model, based on CT images captured from the nail. Because it was difficult to reproduce the fine regions of the shape from the CT images, the screw, blade, and distal locking screw were created using 3D CAD software Fusion360 (Autodesk, Inc., San Rafael, USA). The sizes of the implants are shown in Table 2. Four-node tetrahedral solid elements with minimum and maximum element sizes of 1 and 8 mm, respectively, was used to mesh the inner side of the cancellous and cortical bone. In addition, shell elements with a thickness of 0.3 mm were used on the outer surface of the cortical bone.^22^ To ensure the reliability of the analytical results, a mesh convergence test was performed. Four different models were constructed for each of the two fracture types, with minimum mesh sizes of 0.9, 1, 2, and 3 mm, respectively. The displacement of the femoral head was evaluated when a vertical load of 1500 N was applied, with an increase of <5% as the criterion for convergence. For both basicervical fracture and transcervical shear fracture, the convergence criteria were fulfilled between 2 and 1 mm. However, because of the difficulty in shaping the screw threads at 2 mm, a minimum mesh size of 1 mm was selected. The average number of nodes, shell elements, and solid elements in the FE models were 160204, 73726, and 772690, respectively.

**Table 2:** Size of implants.

Components		Group 1		Group 2		Group 3	
		Short nail	Long nail	Short nail	Long nail	Short nail	Long nail
Lag screw	Length (mm)	90	90	90	90	90	90
	Diameter (mm)	10	10	10.5	10.5	11, 5	11, 5
Nail	Length (mm)	170	260	180	215	170	235
	Diameter (mm)	12	12	11.5	11.5	11	11
Distal locking screw	Length (mm)	40	40	40	40	40	40
	Diameter (mm)	4	4	4	4	4	4

### 2.2 Material properties

**Fig. 3:**
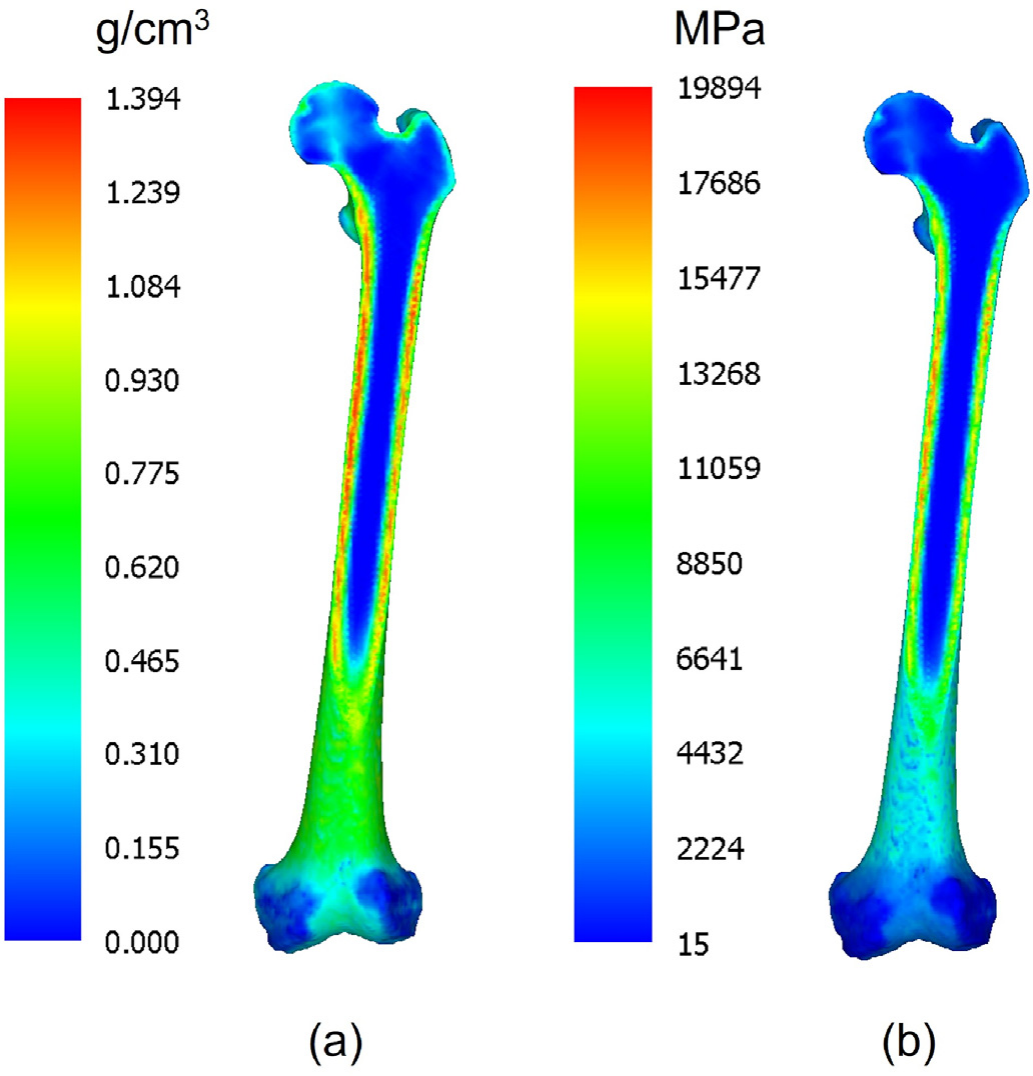
Distribution of (a) bone mineral density and (b) elastic modulus in femur cross-section.

To simulate the subject's specific bone structure, the BMD of each element was determined using Hounsfield unit (HU) values of the CT images (Fig. 3). The relationship between the HU and BMD values was calibrated using a bone mass phantom imaged with the subject's lower extremities to accurately reflect the BMD values in the model. The equation that describes the relationship between the HU value and BMD, i.e., *ρ* (mg/cm^3^), is as follows:

(1) $$\begin{array}{l} \rho =\left\{ \begin{array}{l} \;0.0\;\left(\text{HU}<-1\right)\\ 0.88\times HU-17.1\;\left(\text{HU}\geq -1\right) \end{array}\right.\; \end{array}$$

To determine the apparent Young's modulus *E* (MPa) of each element of the bone, we used the conversion formula proposed by Keyak et al.^23^ as follows:

(2) $$\begin{array}{l} E=\left\{ \begin{array}{l} 0.001\;\left(\rho =0\right)\\ 33900\rho^{2.20}\;\left(0<\rho \leq 0.27\right)\\ 5370\rho +469\;\left(0.27<\rho <0.6\right)\\ 10200\rho^{2.01}\;\left(0.6\leq \rho \right) \end{array}\right. \end{array}$$

The Poisson's ratio of the bone was set to 0.4, and each element was assigned homogeneity.^24^ All implants were assumed to be made of titanium alloy (Ti–6Al–4V), where a Young's modulus of 113.8 MPa,^25^ a Poisson's ratio of 0.34^25^ were assigned to each element homogeneously.

### 2.3 Boundary conditions

**Fig. 4:**
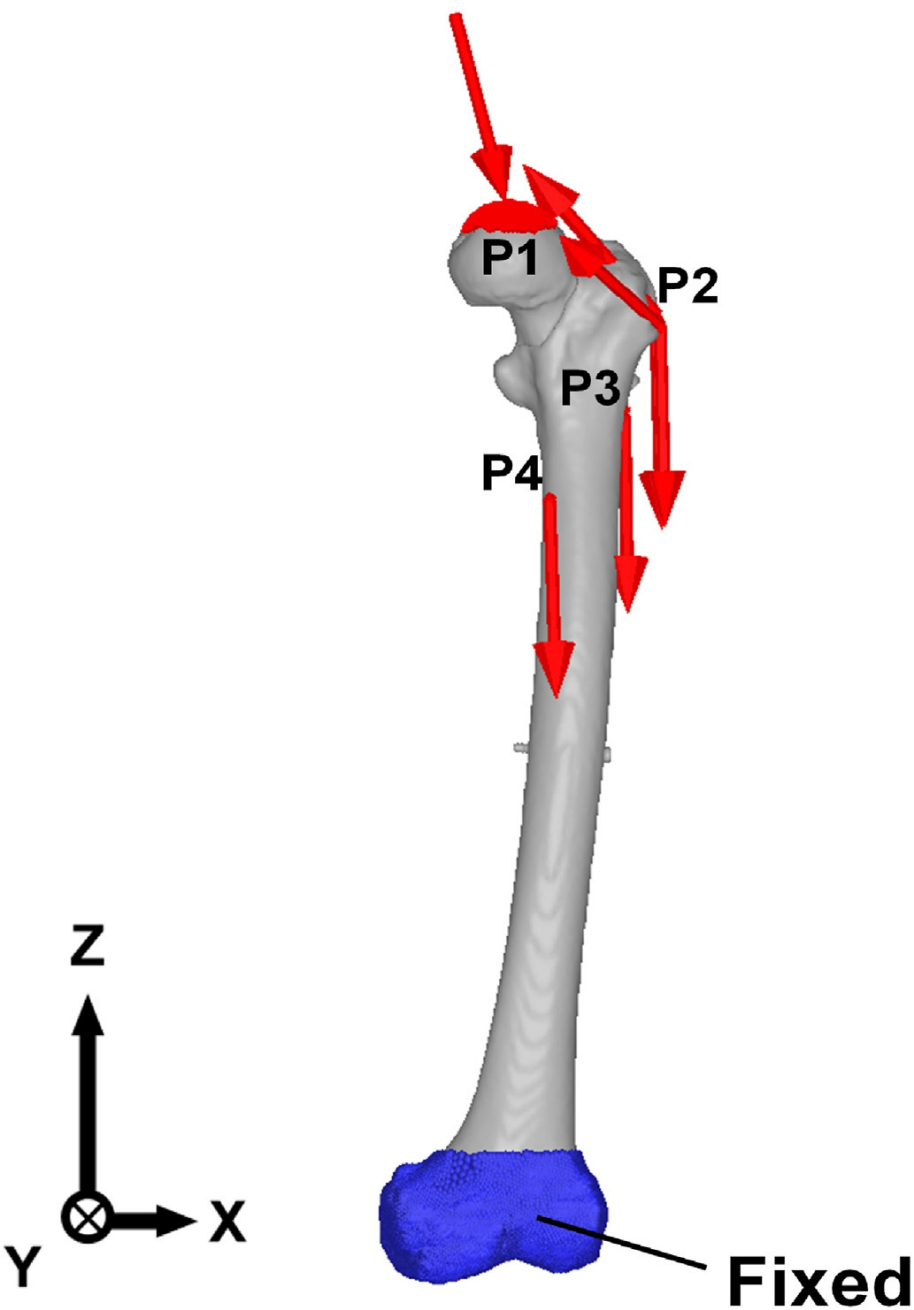
Boundary conditions.

The loading conditions are presented in Table 3. The maximum loads during walking and stair climbing calculated by Heller et al.^26^ were applied to each FE model, as shown in Fig. 4. The load profile included the muscle forces at the femur, normalized by the body weight. Each of the values of the load profile was calculated using the musculoskeletal model and validated using *in vivo* data. To prevent the displacement of the rigid body, the distal end of the femur was fully constrained with six degrees of freedom. The coefficient of friction was 0.3 between the bone and implant,^27^^,^^28^ and 0.46 between bones.^27^^,^^28^ Meanwhile, between-implant components were set as tie conditions (bonded to each other).

**Table 3:** Muscular forces on proximal femur based on walking and stair climbing.^21^^,^^22^

Force	Position	Walking			Stair climbing		
		*F*_*x*_ (N)	*F*_*y*_ (N)	*F*_*z*_ (N)	*F*_*x*_ (N)	*F*_*y*_ (N)	*F*_*z*_ (N)
**Body weight**	P1	0	0	−649	0	0	−649
**Hip contact**	P1	350	213	−1488	386	393	−1534
**Internal resultant**	P1	53	83	−508	84	182	−455
**Abductor**	P2	−376	−28	561	−455	−187	551
**Ilio-tibial tract, proximal part**	P2	–	–	–	−68	−19	83
**Ilio-tibial tract, distal part**	P2	–	–	–	3	5	−109
**Tensor fascia latae, proximal part**	P2	−47	−75	86	−20	−32	19
**Tensor fascia latae, distal part**	P2	3	5	−123	1	2	−42
**Vastus lateralis**	P3	6	−120	−603	14	−145	−877
**Vastus medialis**	P4	–	–	–	57	−257	−1733

### 2.4 Nonlinear fracture analysis

Nonlinear FE analysis was performed to include the yield failure of the trabecular bone elements due to compression. The yield strength in compression was based on the yield criterion proposed by Keyak et al.^29^ The modulus of elasticity after yielding was set to 5% of *E*.^30^ Fracture of the element in compression was defined as the case in which the minimum principal strain of the element was less than −10000.^30^

### 2.5 Selection of evaluation criteria

In this study, the minimum principal strain (compressive strain) and compressive failure element at the fractured fragment (femoral head) were calculated to assess the stability of the fixed fractures. The peri-implant element compressive fracture in the bone fragment (femoral head) was evaluated by assuming a larger volume of the compressive fracture elements.^31^^,^^32^ The larger the volume of the compressive fracture elements, the greater is the risk of displacement of the fractured fragment.

## 3 Results

### 3.1 Minimum principal strain (compressive strain) of head and neck regions

**Fig. 5:**
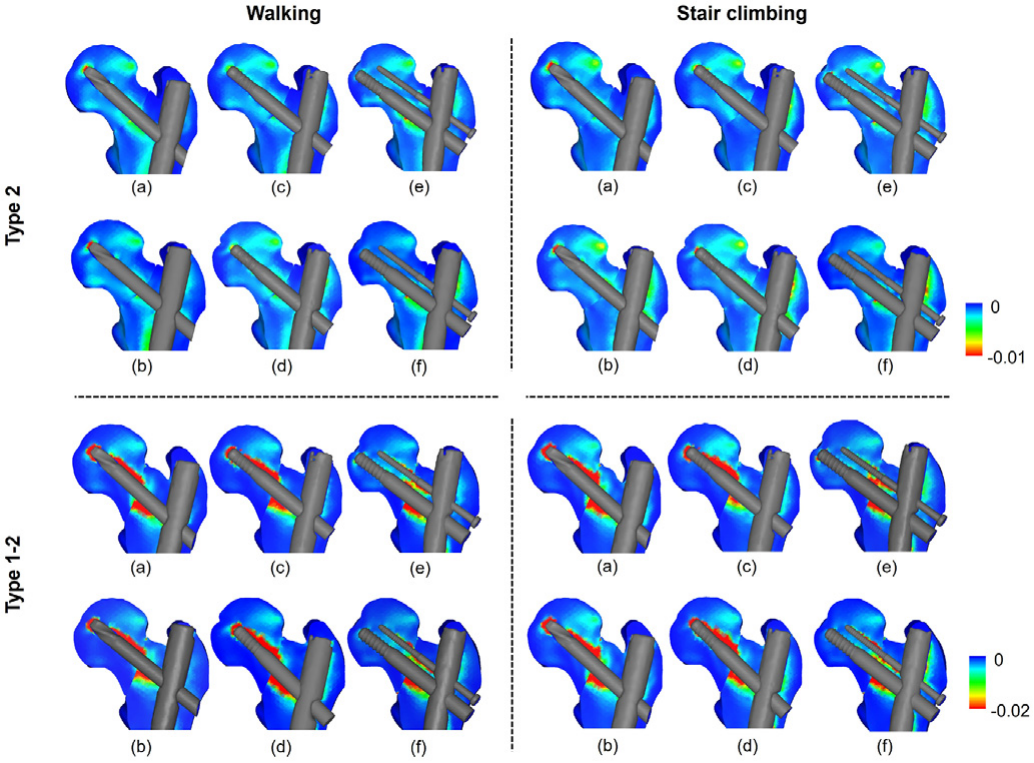
Minimum principal strain distributions of head and neck regions: (a) Short nail of Group 1; (b) long nail of Group 1; (c) short nail of Group 2; (d) long nail of Group 2; (e) short nail of Group 3; (f) long nail of Group 3.

Fig. 5 shows the distribution of the minimum principal strains in the head and neck regions. The bone fragments showed high compressive strain (i.e. lower minimum principal strain) near the screw tip in basicervical fracture and above the screw in transcervical shear fracture. Meanwhile, on the femur side, high compressive strain occurred in the lower region of the screw. It was revealed that the strain site and strain volume differed significantly, although the fracture lines were only slightly different.

### 3.2 Minimum principal (compressive) strain of fracture surface

**Fig. 6:**
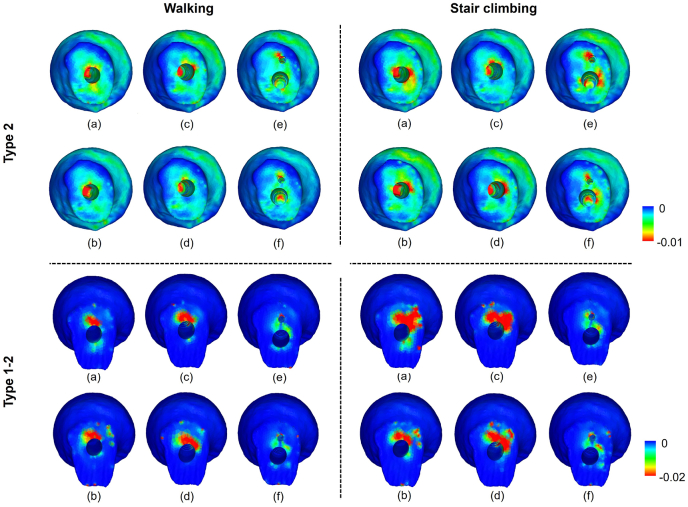
Minimum principal strain distributions on fracture surfaces of fragment (femoral head): (a) Short nail of Group 1; (b) long nail of Group 1; (c) short nail of Group 2; (d) long nail of Group 2; (e) short nail of Group 3; (f) long nail of Group 3.

Fig. 6 shows the distribution of the minimum principal strain on the fracture surface. In both the basicervical fracture and transcervical shear fracture, the highly compressive strain (i.e. lower minimum principal strain) tended to be concentrated superior to the screw because of the shearing force at the fracture regions. Fig. 8 shows the minimum value of the minimum principal strain (i.e. maximum compressive strain) on the fracture surface. The short nails showed lower values than the long nails for both basicervical and transcervical shear fractures. Comparing the average values of walking and stair climbing, basicervical fracture showed the lowest value (−0.018) for Group 2 with a short nail and the highest value (−0.012) for Group 3 with a long nail. Conversely, transcervical shear fracture showed the lowest value (−0.288) for Group 1 with a short nail and the highest value (−0.162) for Group 3 with a long nail.

### 3.3 Compressive fracture element at fragment

**Fig. 7:**
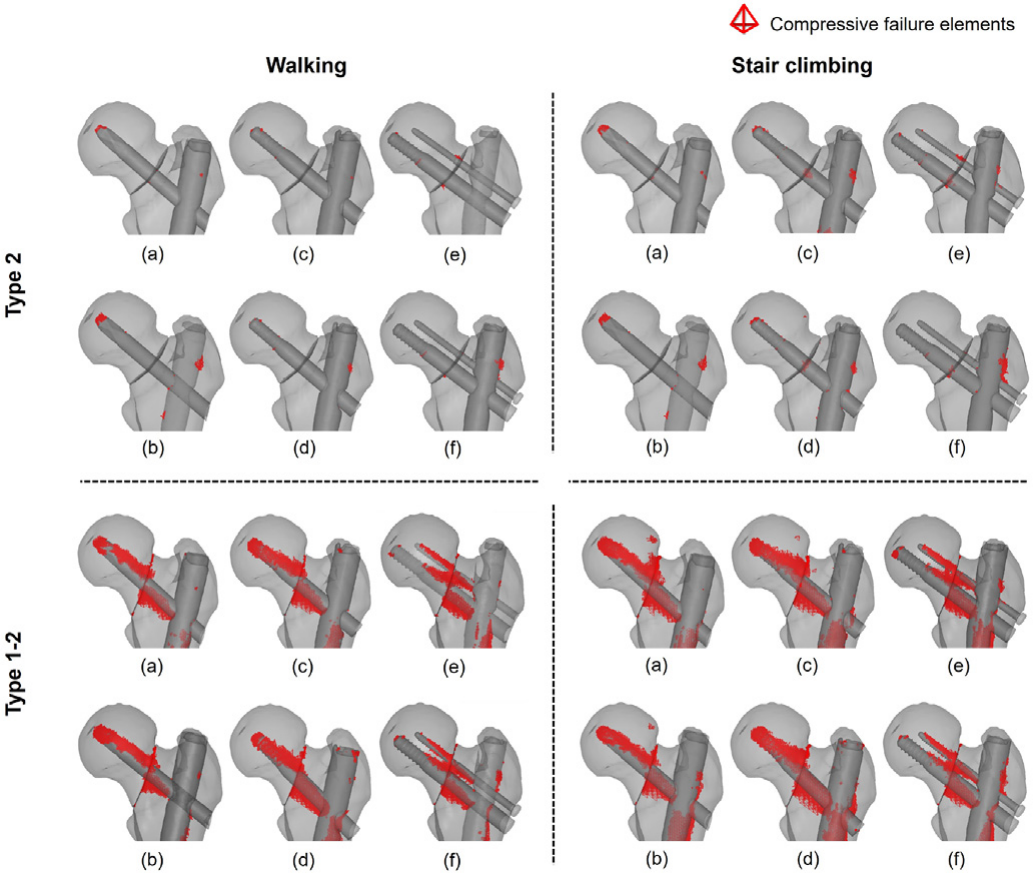
Distribution of compressive failure elements: (a) Short nail of Group 1; (b) long nail of Group 1; (c) short nail of Group 2; (d) long nail of Group 2; (e) short nail of Group 3; (f) long nail of Group 3.

The region where compressive failure occurred was the same as the region where a high compressive strain occurred (Fig. 5, Fig. 7). The total volume of the compressive failure elements was larger for stair climbing than for walking (Fig. 9). In basicervical fracture, the minimum volume of the compressive failure elements was recorded in Group 3 with a long nail (2 mm^3^), where the maximum was recorded in Group 1 with a long nail (35 mm^3^). Meanwhile, in transcervical shear fracture, the minimum was recorded in Group 3 with a long nail (217 mm^3^), whereas the maximum was recorded in Group 2 with a short nail (1169 mm^3^).

**Fig. 8:**
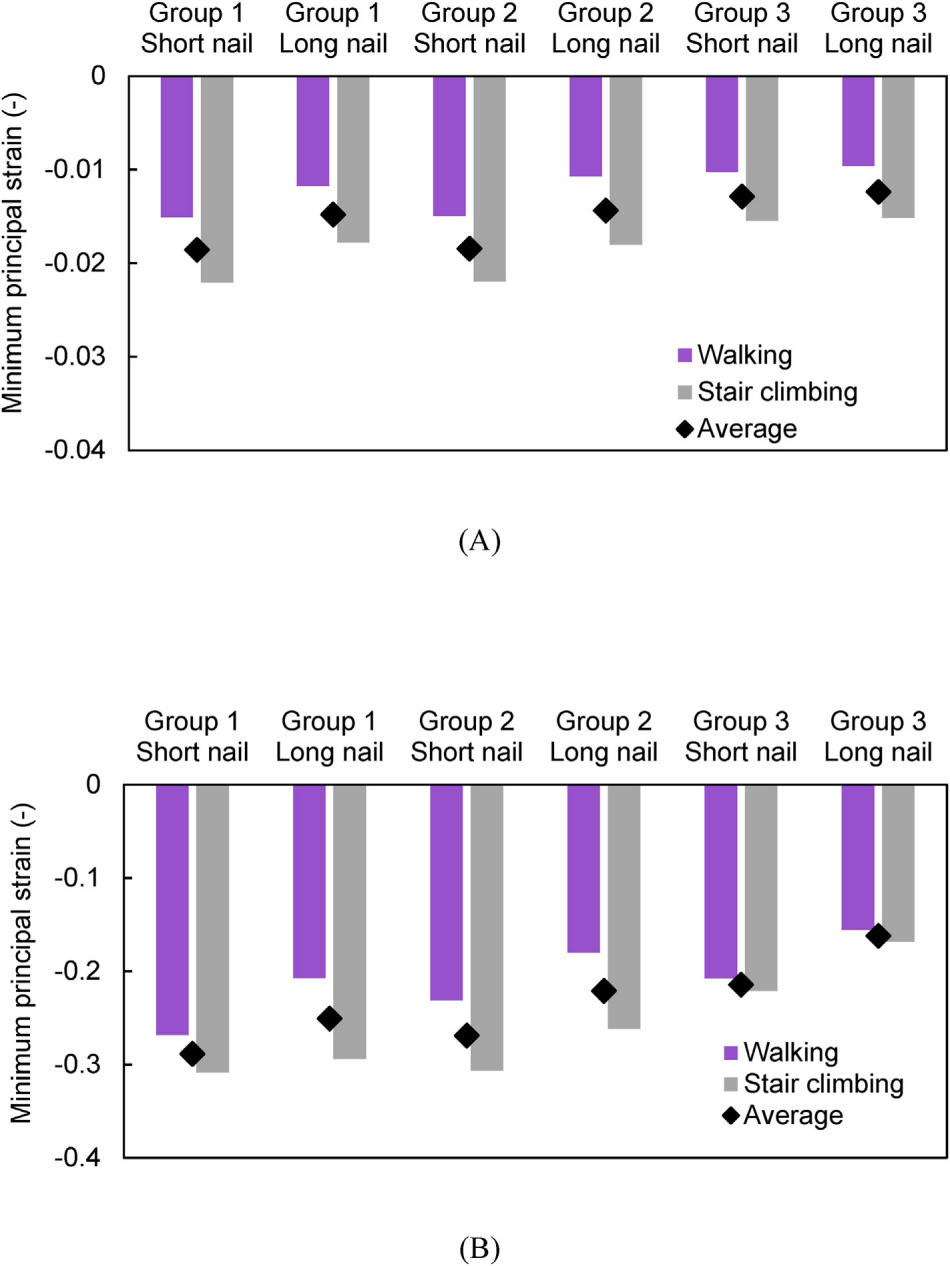
Minimum value of minimum principal strain at each fracture surface: (A) Basicervical fracture; (B) transcervical shear fracture.

**Fig. 9:**
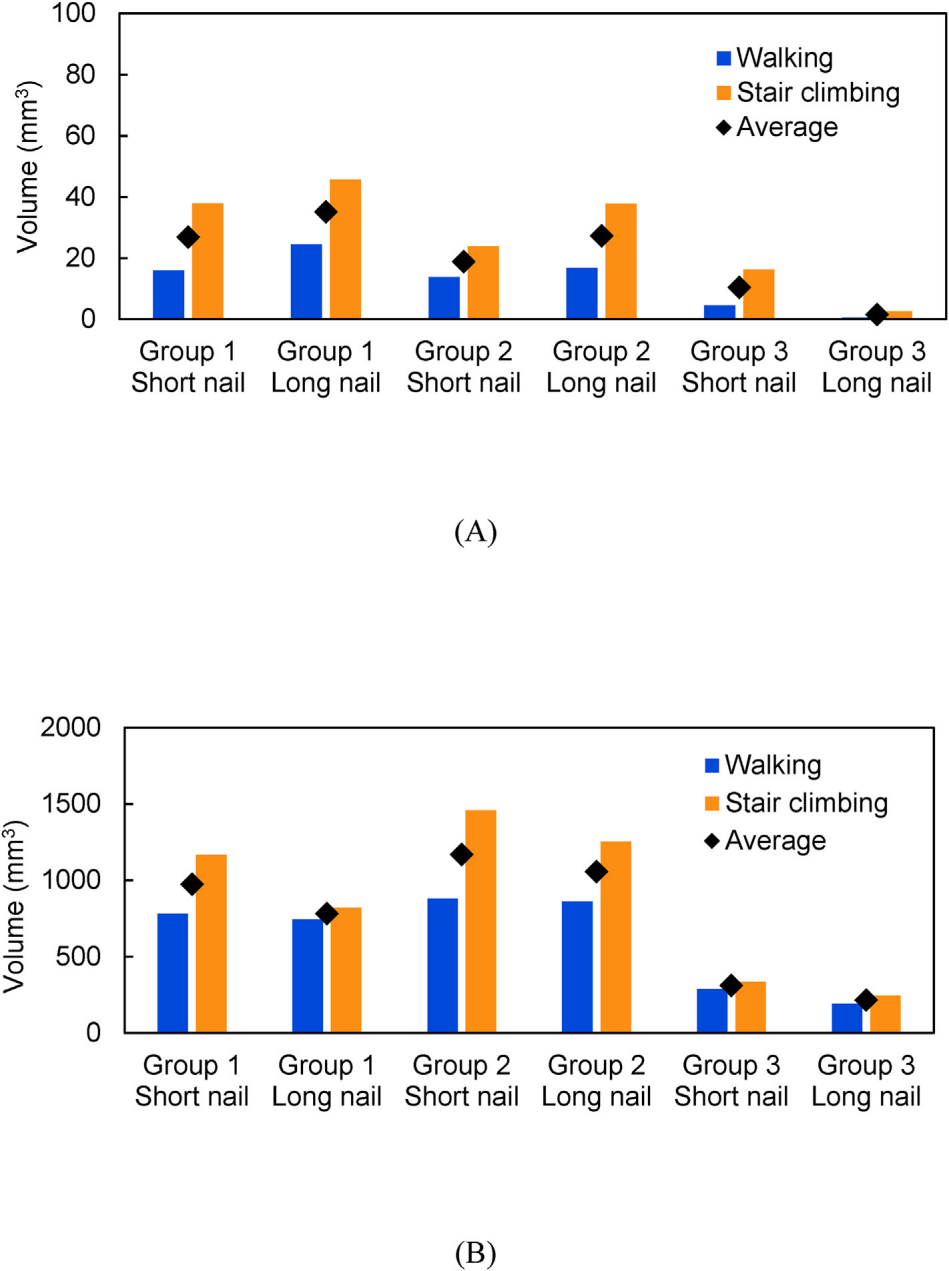
Volume of compressive failure elements in (A) Basicervical fracture and (B) transcervical shear fracture.

## 4 Discussion

Low BMD is a significant factor that increases the risk of failure after surgery.^33^ To achieve better fixation for osteoporotic proximal femoral fractures, an optimal device must be selected.^14^ In this study, we compared the fixation of six different intramedullary implants for two types of fractures (basicervical and transcervical shear fractures) using an osteoporotic bone model. Our findings indicated that long nails with double lag screws afforded the best fixation for both fractures. Furthermore, the long nail afforded better fixation against shearing forces in the fracture than the short nail. The results of this study are valuable for the selection of treatments for two types of unstable fractures.

It was discovered that the compressive strain at the fracture site was advantageous to the bone healing process; however, Peter et al.^34^ reported that the shearing force at the fracture site was a disadvantage. In the present analysis, a high compressive strain occurred superior to the screw in the femoral head, and a high compressive strain occurred inferior to the screw on the femoral side (Fig. 5). This indicates that a high compressive strain was generated around the screw owing to the shearing force in the fracture region (Fig. 6). Furthermore, excessive compressive strain resulted in the microfracture of the trabecular bone, which resulted in complications such as secondary displacement and cut-out.^31^^,^^33^ This suggests that the compressive strain in the fracture surface represents the fixation against the force in the shear direction of the fracture region.

Kwak et al. reported that fixation achieved using a blade and screw yielded better outcomes as compared with fixation using a single blade for basicervical fracture.^14^ Our results showed that better fixation was achieved using double lag screws compared with using single lag screws for both basicervical and transcervical shear fractures (Fig. 3, Fig. 4). In both fractures, shearing forces were generated at the fracture surface when a load was applied. Therefore, it is suggested that the compressive force generated around the screw is more dispersed in two devices than in the case of one device that pierces the femoral head, i.e. the implant with a single lag screw or single blade. This tendency was confirmed by the fact that the difference in the volume of compressive fracture between the loading conditions was smaller when two devices were inserted into the femoral head than when one device was inserted.

Previous studies reported that better fixation was obtained by increasing the intramedullary nail length in various fracture types.^35^^,^^36^ In also basicervical and transcervical shear fractures, which are known to be highly unstable, long nails offered better fixation than short nails, and this tendency is more evident in transcervical shear fractures. The distribution of the minimum principal strain showed that the red area of the large compressive strain (Fig. 3) was concentrated in the upper region of the lag screw, and this tendency was more evident in the transcervical shear fracture. Generally, femoral neck fractures are more unstable as the main fracture line becomes vertical.^37^ Our results show that transcervical shear fracture, whose main fracture line is more vertical, is more unstable than basicervical fracture. This finding supports common understanding. In addition, comparing the volume of compressive failure elements in each implant, the maximum value was approximately 22 and 5 times higher than the minimum values in the basicervical fracture and transcervical shear fracture, respectively. This indicates the importance of selecting an optimal device.

This study has several limitations. First, the number of participants was small. Further studies should be conducted to investigate the effects of changes in bone shape and quality. Second, the contact area between the implant components was fixed. However, in reality, because a sliding mechanism exists in the contact area, friction in the contact area should be defined and analysed. Third, only the main fracture line was applied to each finite element model. In fact, bone fragments were generated at the fracture site.^38^ The reproduction of fractures will be investigated in future studies.

## 5 Conclusions

The present study showed that a long intramedullary nail with double lag screws was the most effective implant for the fixation of proximal femoral basicervical fracture (AO 31-B3, Type 2 in area classification) and transcervical shear fracture (AO 31-B2.3, Type1-2 in area classification). In addition, it was proven for the first time that transcervical shear fracture is a significantly more unstable fracture than basicervical fracture.

## Declarations of competing interest

None.
